# Laser therapy for onychomycosis in patients with diabetes at risk for foot complications: study protocol for a randomized, double-blind, controlled trial (LASER-1)

**DOI:** 10.1186/s13063-015-0622-4

**Published:** 2015-03-22

**Authors:** Leonie Nijenhuis-Rosien, Nanne Kleefstra, Maurice J Wolfhagen, Klaas H Groenier, Henk JG Bilo, Gijs WD Landman

**Affiliations:** Diabetes Centre, Isala, Dr Spanjaardweg 11, 8025 BT Zwolle, the Netherlands; Innofeet Voetencentrum Nijenhuis Podiatry, Simon Stevinweg 13, 8013 NA Zwolle, the Netherlands; Department of Internal Medicine, University of Groningen, University Medical Center Groningen, Hanzeplein 1, 9700 RB Groningen, the Netherlands; Langerhans Medical Research Group, Postbus 21, 4254 ZG Sleeuwijk, the Netherlands; Department of Medical Microbiology, Isala, Dr. van Heesweg 2 8025 AB Zwolle, the Netherlands; Department of General Practice, University of Groningen, University Medical Center Groningen, Hanzeplein 1, 9700 RB Groningen, the Netherlands; Department of Internal Medicine, Gelre Hospital, Albert Schweitzerlaan 31, 7334 DZ Apeldoorn, the Netherlands

**Keywords:** Onychomycosis, Nd:YAG laser, Diabetic foot, Diabetic foot ulcer

## Abstract

**Background:**

In a sham-controlled double-blind trial, we aim to establish the efficacy and safety of the local application of laser therapy in patients with diabetes, onychomycosis and risk factors for diabetes-related foot complications. Onychomycosis leads to thickened and distorted nails, which in turn lead to increased local pressure. The combination of onychomycosis and neuropathy or peripheral arterial disease (PAD) increases the risk of developing diabetes-related foot complications. Usual care for high-risk patients with diabetes and onychomycosis is completely symptomatic with frequent shaving and clipping of the nails. No effective curative local therapies exist, and systemic agents are often withheld due to concerns for side effects and interactions.

**Methods/Design:**

The primary aim is to evaluate the efficacy of four sessions of Nd:YAG 1064 nM laser application on the one-year clinical and microbiological cure rate in a randomized, double-blind, sham-controlled design with blinded outcome assessment. Mandatory inclusion criteria are diagnosis of diabetes, risk factors for developing foot ulcers defined as a modified Simm’s classification score 1 or 2 and either neuropathy or PAD. A total of 64 patients are randomized to intervention or sham treatment performed by a podiatrist.

**Discussion:**

This study will be the first double-blind study that investigates the effects of local laser therapy on onychomycosis, specifically performed in patients with diabetes with additional risk factors for foot complications.

**Trial registration:**

Clinical trials.gov as NCT01996995, first received 22 November 2013.

## Background

The prevalence of onychomycosis is 2.5 to 2.8 times higher in patients with diabetes [1-3]. The combination of onychomycosis with other risk factors, like neuropathy, increases the risk of developing diabetes-related foot complications [4]. Onychomycosis leads to thickening and sharpening of the nails. Thickening of the nails potentially increases subungual pressure and compromises vascular flow [5,6]. Sharpening of the nails can lead to small skin injuries, which are potential portals of entry for pathogens. In the presence of neuropathy and/or peripheral arterial disease (PAD), onychomycosis is an additive risk factor for diabetes-related foot complications like foot ulcers, cellulitis, osteomyelitis and gangrene [7-10]. Worldwide, foot ulcers in patients with diabetes are the leading cause of hospitalizations and amputations [11-15]. The development of foot ulcers has been associated with considerable disability [16-18], and the presence of onychomycosis has been associated with various emotional and social problems [19-23].

Treating onychomycosis is increasingly recognized as a potential strategy for preventing diabetes-related foot complications [5,24-26]. As part of the national Dutch foot care program, usual care for patients with onychomycosis is symptomatic; every six weeks, nails are skived to normal proportions and sharp edges are clipped and removed. This podiatric treatment aims to prevent ulcer formation and the development of infections through reducing subungual pressure and sharpen nails. Although effective systemic antifungal agents are available, they are often withheld due to concerns of side effects and interactions [27]. Furthermore, studies that investigated antifungal agents mostly excluded patients with diabetes. Despite many claims of efficacy, no proven effective local therapies exist. An effective and safe curative treatment option for onychomycosis could lead to a decrease in diabetes-related foot complications, especially in patients with other risk factors. By reducing the frequency of ‘skiving and clipping’ treatment sessions and by reducing the development of ulcers and infections caused by treatment-resistant onychomycosis, local therapies could become valuable preventive treatment option [28].

Local laser therapy is becoming increasingly popular as a treatment modality for a variety of dermatologic conditions and is being applied in the treatment of onychomycosis [29]. The use of lasers for improving toenail appearance has become one of the most rapidly approved therapies by the US Food and Drug Administration (FDA) [29]. Unlike pharmacologic agents, laser systems are presumed to have predictable adverse effects [29]. There are few nonrandomized trials and very few randomized studies investigating effect of laser therapy on onychomycosis. None had a double-blind design, and none were performed in patients with diabetes who also had additional risk factors for foot ulcers [29].

The primary aim of this trial is to evaluate the efficacy of four sessions of N-YAG 1064nM laser application on the one-year complete cure rate of onychomycosis in patients with diabetes and risk factors for developing diabetes related foot complications. Secondary outcomes are microbiologic cure rate and improvement in health-related quality of life (HRQOL).

## Methods/Design

Primary and secondary care patients with type 1 and type 2 diabetes mellitus with risk factors for developing diabetic foot ulcers and a clinical suspicion of onychomycosis will be recruited from a single-center podiatry practice. Patients will be included after written informed consent and microbiologic confirmation of onychomycosis.

Nail dust is collected from the target nail for microbiologic confirmation with blankophor microscopy, culture and polymerase chain reaction (PCR). PCR is regarded as the gold standard. The PCR is a real-time multiplex PCR based on amplification of ribosomal internal transcribed spacer regions and identification by probes specific for *Trichophyton mentagrophytes* species complex, *Trichophyton tonsurans*, *Trichophyton violaceum*, *Trichophyton rubrum* species complex, *Microsporum canis*, *Microsporum audouinii* and *Epidermophyton floccosum* as described by Arabatzis *et al.* [30].

The target nail is the nail with the highest onychomycosis severity index. The severity of onychomycosis is evaluated with the onychomycosis severity index (OSI) and standardized photographs are made [31]. Evaluation of treatment results will be done by an independent expert panel blinded for treatment allocation. The three experts are experienced health care providers who have experience in the treatment of patients with diabetic foot problems. Health-related quality of life (HRQOl) is evaluated at baseline and after follow-up with a generic questionnaire, the WHO-5, and a disease specific questionnaire the NailQOl [23]. The trial has received research ethics board approval from METC Isala Zwolle (NL46084.075.13/METC nr 13.0885).

### Study endpoints

The primary endpoint is complete cure rate of the target nail. Complete cure is defined as a completely normal nail, or negative microbiological results in case minor abnormalities are present. A minor abnormality is defined as an irregularity of the nails with less than 5% of the surface area of the target nail at less than 1/4 of the distance of the distal nail edge without hyperkeratosis. Secondary endpoints are microbiologic cure rate of the target nail, complete clinical cure of all affected toes, complete clinical cure of the target nail, markedly clinically improvement of the target nail, onychomycosis severity index below 6 (in patients with scores >6 at study entry) of the target nail, changes in the affected surface of the target nail, changes in affected surface of all nails with a clinical diagnose of infection, free of subungual hyperkeratosis and changes in HRQOL. A ‘markedly clinical nail improvement’ is defined as a nail with less than 10% abnormalities and without hyperkeratosis after 52 weeks.

### Patient selection criteria

**Figure 1:**
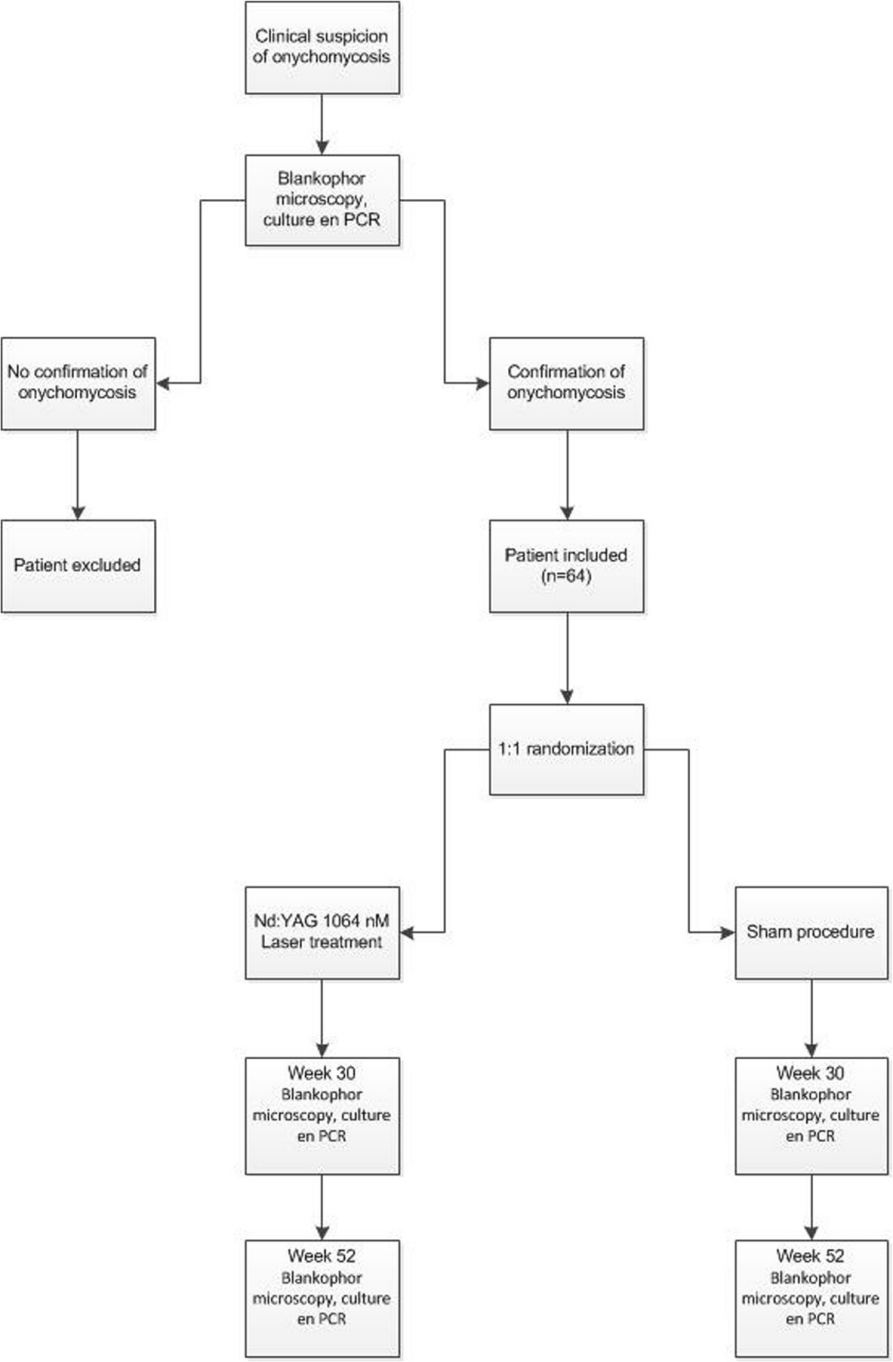
**Study flow chart.**

Patients with clinical suspicion and microbiologic confirmation of onychomycosis are randomized to laser treatment or sham, see Figure 1.

Mandatory inclusion criteria are diagnosis of T1DM or T2DM, 18 years or older, at risk for diabetic foot ulcers defined by a modified Simm’s classification score 1 or 2 with either neuropathy or PAD [32], and nail involvement of at least 25% of the target nail [3]. See Table 1 for the modified Simm’s classification.

**Table 1:** **The modified Simms’ classification**

**Simm’s classification**	**Risk profile**	**Control frequency**
Simm’s 0	No loss of PS or PAV	Once in 12 months
Simm’s 1	Loss of PS or PAD, Without signs of local increased pressure	Once in 6 months
Simm’s 2	Loss of PS and PAD. Loss of PS and/or PAD in combination with signs of local increased pressure.	Once in 3 months
Simm’s 3	Ulcer or amputation in the medical history	Once in 1 to 3 months

PAD, peripheral arterial disease; PS, protective sensibility.

Exclusion criteria are no microbiologic confirmation, Simms’ classification score 3, the presence or history of diabetic foot ulcers, ischemic pain, ankle brachial index <0.9, patients receiving dialysis, severe renal insufficiently (eGFR below 30 ml/min), a documented toe pressure below 50 mmHg, use of systemic or topical antifungal agents 3 months prior to inclusion, use of immunosuppressive drugs, presence of psoriasis, lichen planus, or other abnormalities that could result in clinically abnormal toenails, a history of epilepsy and insufficient knowledge of the Dutch language. Patients with a dark skin color (Fitzpatrick 4 and 5) are excluded since dark skin color is associated with dark nails, which theoretically lead to increased temperatures during laser application [33].

### Treatment assignment

Patients will be randomized in blocks using sealed nontransparent envelopes to either laser or sham treatment. A cloth between the head and his or her feet and a blinded goggle (which also protects the eyes) is used for blinding the subjects. The periprocedural sounds and lights during the laser application and the sham procedure are identical. The investigators are blinded for treatment allocation through the use of an independent second podiatrist who performs the interventions. An independent three-person panel blinded for treatment allocation evaluates the study outcomes using standardized photographs made at baseline, week 30 and at the end of follow-up. When consensus cannot be reached, decisions are made through majority voting.

### Clinical follow-up

Patients are treated with laser session in week 0, 2, 4, and 12. The settings are*;* 1064 nm, spot size 3 mm, 20 J /cm2, 5 Hz, power 10 W. A maximum of two sequential sessions (one session on the horizontal and one the vertical passing) will be applied to eliminate potential safety issues in those patients with a lack protective sensibility.

After 52 weeks, complete regrowth of the nail can be expected. At baseline, 30 and 52 weeks all target nails are sampled for blankophor microscopy, culture and polymerase chain reaction.

At baseline, 30 and 52 weeks, all nails are evaluated using the following scale: completely healed, markedly improved (less than 10% affected), minor improvement, unchanged, and worse, and with the onychomycosis severity index [31]. The microbiological outcomes from week 30 are used to differentiate re-infection from persistent infection. Patients with re-infection have negative microbiological results at week 30 and positive results at week 52. Patients in whom different organisms at baseline and week 30 or 52 are isolated are regarded as having a reinfection. Patients are regarded as having a persistent infection when baseline, week 30 and week 52 microbiological results provide evidence of the same organism. Week 52 results, including the microbiological results, are used for evaluation of the primary and secondary outcomes. See Table 2 for the follow-up survey.

**Table 2:** **Follow-up survey**

	**Baseline**	**Week 0**	**Week 2**	**Week 4**	**Week 12**	**Week 30**	**Week 52**
		**Session 1**	**Session 2**	**Session 3**	**Session 4**		
Inclusion		x					
Randomization		x					
Blankophor microscopy/Culture	X					x	X
/PCR							
Photo	X				x	x	X
Treatment		x	x	x	x		

### Data analysis

Estimating the proportion of patients with a complete cure rate to be 40% in the intervention group and 5% in the control group [27], with a power of 0.85, an alpha of 0.05 (2-tailed), the sample size should be at least 56. Accounting for loss to follow-up, the total sample size will be aimed at 64.

Data entry is done in duplicate by persons not otherwise involved in the study. All statistical analyses are carried out by a statistician blinded for treatment allocation. The Fisher’s Exact test will be used to test for differences between the treatment groups, and 95% confidence intervals (CIs) will be constructed for the differences in clinical and microbial cure rates. Normally distributed continuous variables will be compared using the Student *t*-test, and non-normally distributed variables will be compared with the Mann-Whitney *U* test. A significance level of less than 5% is regarded as significant. Analyses will be performed according to the intention-to-treat principle. Separate per-protocol analyses are planned in patients in whom all treatment sessions are performed and for analyses excluding re-infection. A sensitivity analysis is planned that regards completely cured patients at week 30 who have a new infection at week 52 as having a complete cure. All endpoints are evaluated at week 52.

## Discussion

Patients with diabetes who suffer from onychomycosis and neuropathy or PAD have an increased risk for developing diabetes-related foot complications, but also for treatment-related complications. New, effective and safe treatment modalities are needed for patients with diabetes who have risk factors for diabetes-related foot complications, in order to minimize the risks of developing ulcers and also to minimize discomfort and treatment-related complications caused by the current usual care.

The results of this study will provide the first double-blinded evidence for laser therapy and the first study in patients with diabetes mellitus with risk factors for developing diabetic foot ulcers. Positive results could aid in the prevention of diabetes-related foot complications since no local curative options are available, and systemic treatment could be avoided. If the results were to show safety and efficacy of laser therapy, this would provide the first high-level evidence for efficacy of a local treatment modality against onychomycosis. The results of this study will be at least equally relevant if they were to show non-efficacy since the use of lasers is becoming increasingly popular. In that case, unnecessary laser applications and possible harm could be avoided.

## Trial status

Recruiting.

## Abbreviations

CI: confidence intervals
FDA: Food and Drug Administration
HRQOL: health-related quality of life
OSI: Onychomycosis Severity Index
PAD: peripheral arterial disease
PCR: polymerase chain reaction
QoL: quality of life
